# Supplementary material for: Lymphaticovenous Anastomosis for Age-Related Lymphedema
Source: J Clin Med. 2021 Oct 31;10(21):5129. doi: 10.3390/jcm10215129 (PMC8584733; doi:10.3390/jcm10215129)
Supplement: Supplementary file 1 [file jcm-10-05129-s001.zip › jcm-1422532-supplementary.pdf]

Supplementary Materials

Table S1. The number of cases excluded from the study.

	**<35 Group**	**35–64 Group**	**≧65 Group**	**Sum Number**
Initial number for screen	20	204	266	**490**
Number of cases excluded from the study				
Need for support	0	5	43	**48**
Body mass index (BMI) ≥35	0	15	7	**22**
History of a major invasive procedure to pelvic cancer	2	132	96	**230**
Radiation therapy to the lower limbs or abdomen	0	2	3	**5**
Heart failure	0	0	2	**2**
Renal failure	0	1	1	**2**
Cirrhosis of the liver,	0	1	2	**3**
Hypoproteinemia	0	5	7	**12**
Deep vein thrombosis	0	2	8	**10**
Chronic venous obstruction or venous reflux	0	12	26	**38**
Thyroid dermopathy	0	2	5	**7**
Other endocrine cause of edema	0	3	2	**5**
Drug-induced edema	1	5	9	**15**
Arterial and/or venous malformation;	1	4	0	**5**
Total number of cases excluded from the study	4	189	211	404
Total number of cases included into this study	16	15	55	86

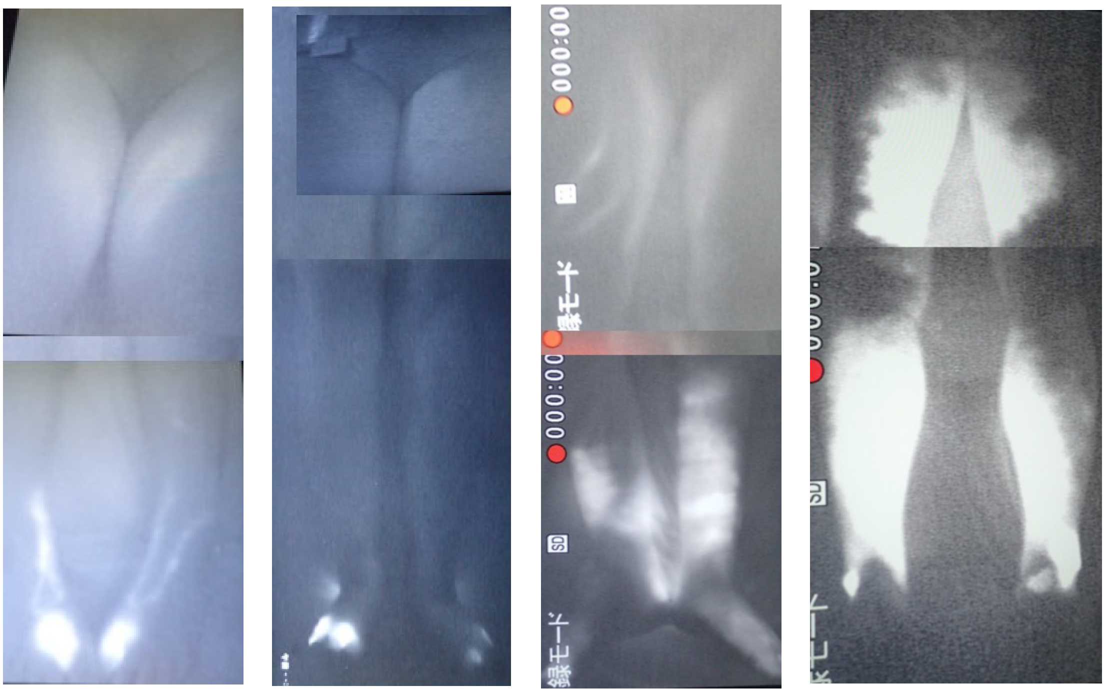

Figure S1. Patterns of age-related primary lymphedema in the lower extremities seen on indocyanine green lymphography.

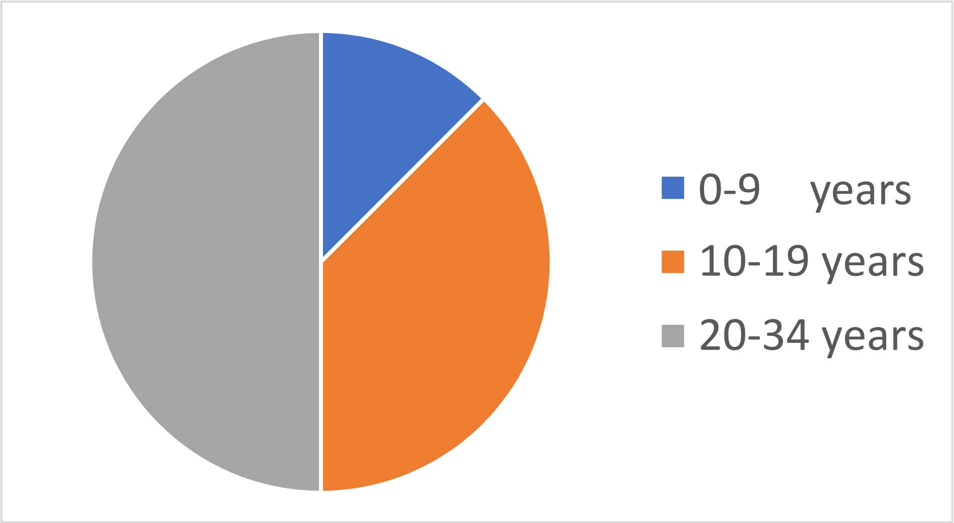

Figure S2. Number of cases divided by its onset age in <35 group.

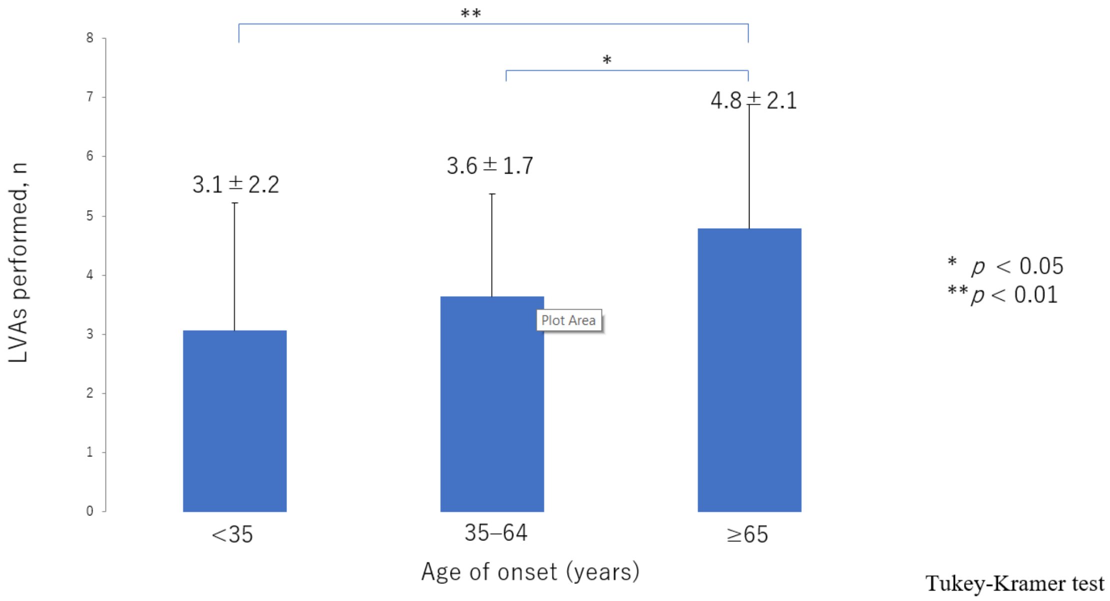

Figure S3. Number of performed LVAs according to age of onset.

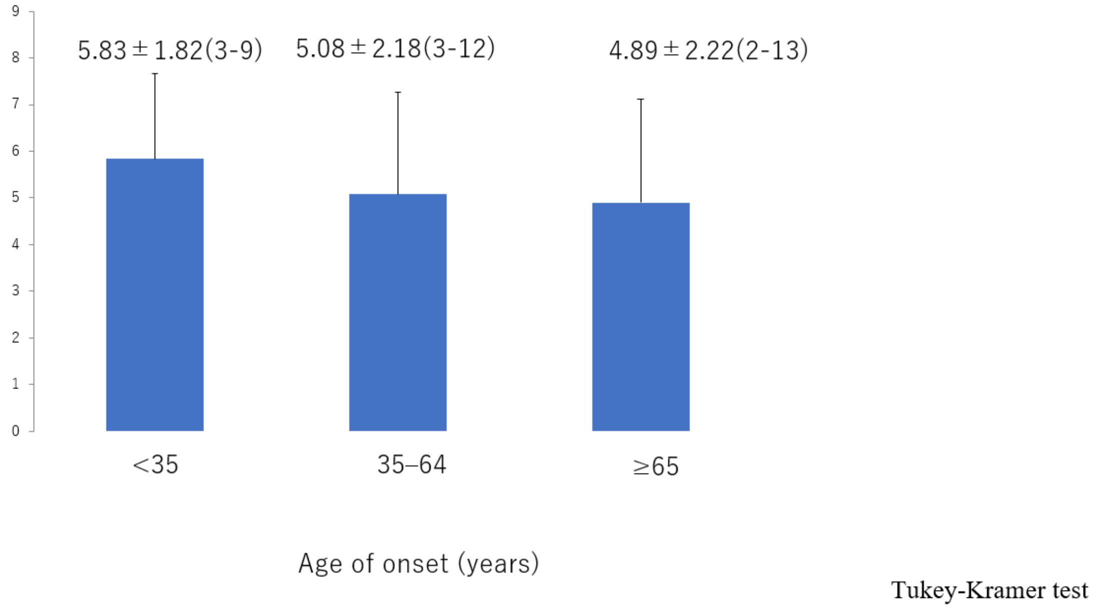

Figure S4. Number of skin incisions for LVA.

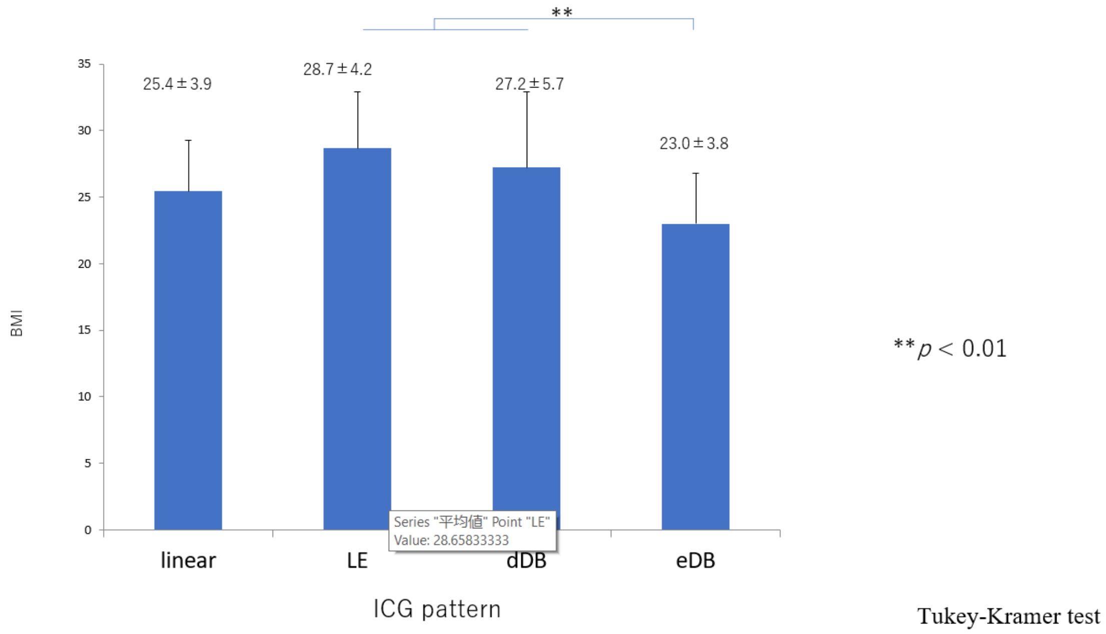

Figure S5. ICG pattern according to BMI independent of age.
